# *Cutibacterium acnes* (formerly* Propionibacterium acnes*) isolated from prosthetic joint infections is less susceptible to oxacillin than to benzylpenicillin

**DOI:** 10.7150/jbji.30954

**Published:** 2019-04-20

**Authors:** Sara Ridberg, Bengt Hellmark, Åsa Nilsdotter, Bo Söderquist

**Affiliations:** 1 School of Medical Sciences, Faculty of Medicine and Health, Örebro University, Örebro, Sweden; 2 Department of Infectious Diseases and Department of Clinical and Experimental Medicine, Linköping University, Norrköping, Sweden

**Keywords:** *Cutibacterium acnes*, *Propionibacterium acnes*, prosthetic joint infections, antibiotic susceptibility testing, antibiotic prophylaxis

## Abstract

**Introduction:** The frequency of prosthetic joint infections (PJIs) due to *Cutibacterium acnes* (formerly* Propionibacterium acnes*) is increasing, especially shoulder PJIs. The recommended antibiotic prophylaxis for hip and knee arthroplasties is beta-lactam antibiotics, predominantly cephalosporins. However, for example in Sweden, isoxazolyl-penicillin cloxacillin is used. No specific recommendations for shoulder arthroplasties are available. The aim of the present study was to determine the minimum inhibitory concentration (MIC) values for different antibiotics for *C. acnes*; and, more specifically, to compare the MIC values for benzylpenicillin and oxacillin.

**Materials and methods:** Minimum inhibitory concentration (MIC) values for nine different antibiotic agents were obtained by gradient test (Etest) using strains of *C. acnes* (n= 57) isolated from PJIs from shoulders (n=31), hips (n=21), and knees (n=5).

**Results:** All isolates had low MIC values for most of the tested antibiotic agents, and showed a wild type MIC distribution. The exception was clindamycin with 9% of the isolates displaying decreased susceptibility. The MIC values obtained for benzylpenicillin were significantly lower than the MIC values for isoxazolyl-penicillin (oxacillin).

**Conclusion:** These *in vitro* results indicate that benzylpenicillin might be a more effective prophylactic treatment to prevent shoulder PJIs caused by *C. acnes*. However, further studies on the subject are needed, and the effectiveness of the prophylactic treatment should be evaluated using randomized controlled studies and/or register-based studies.

## Introduction

Infections associated with orthopedic joint implants, such as prosthetic joint infections (PJIs), are a major threat to patients' quality of life following prosthetic joint surgery. Perioperative antibiotic prophylaxis is universally used to reduce the risk of surgical site infections 1. In many countries, various cephalosporins are used as first-line prophylaxis 2. However, in Sweden, isoxazolyl-penicillin cloxacillin is recommended as antibiotic prophylaxis for all primary joint replacements [PRISS Expert Group 2; http://lof.se/patientsakerhet/vara-projekt/rekommendationer/].

The most common microorganisms that cause PJIs are staphylococci 2,3. Anaerobic bacteria, predominantly *Cutibacterium acnes* (formerly* Propionibacterium acnes*), cause less than 5% of PJIs 4,5,6,7. However, *C. acnes* has been reported to be responsible for >50% of infections following shoulder surgery 7,8. Therefore, at centers for shoulder surgery in Sweden, the recommended antibiotic prophylaxis regimens have been reviewed. Cloxacillin was replaced by clindamycin or supplemented by adding benzylpenicillin (personal communication).

The aim of the present study was to determine the minimum inhibitory concentration (MIC) values for different antibiotics against *C. acnes* isolated from patients with PJIs; and, more specifically, to compare the MIC values for benzylpenicillin and oxacillin in order to determine if a change in the existing antibiotic prophylactic regimen for shoulder arthroplasties is justified.

## Materials and Methods

*C. acnes* (n=57) isolated from orthopedic implant-associated infections mainly PJIs were obtained from the Departments of Clinical Microbiology at the University Hospitals of Örebro and Linköping from 2002 to 2015. Isolates came from shoulder (n= 31), hip (n= 21), and knee (n= 5) orthopedic implant-associated infections.

The antibiotic susceptibility pattern was tested for nine different antibiotics; benzylpenicillin, oxacillin, ampicillin, amoxicillin, cefuroxime, clindamycin, rifampicin, daptomycin, and vancomycin. MIC values were determined by Etest (bioMérieux, Marcy -l'Étoile, France). The *in vitro* antibiotic susceptibility testing was performed on FAA plates (4.6% LAB 90 fastidious anaerobe agar; LAB M, Heywood, United Kingdom, supplemented with 5% (vol/vol) horse blood) with 0.5 McFarland suspensions of bacteria in NaCl and incubation at 36°C under anaerobic conditions for 1 day.

### Ethics

This study used bacterial isolates from humans. No tissue material or other biological material was stored from the patients. All information regarding these isolates was anonymized.

## Results

**Figure 1:**
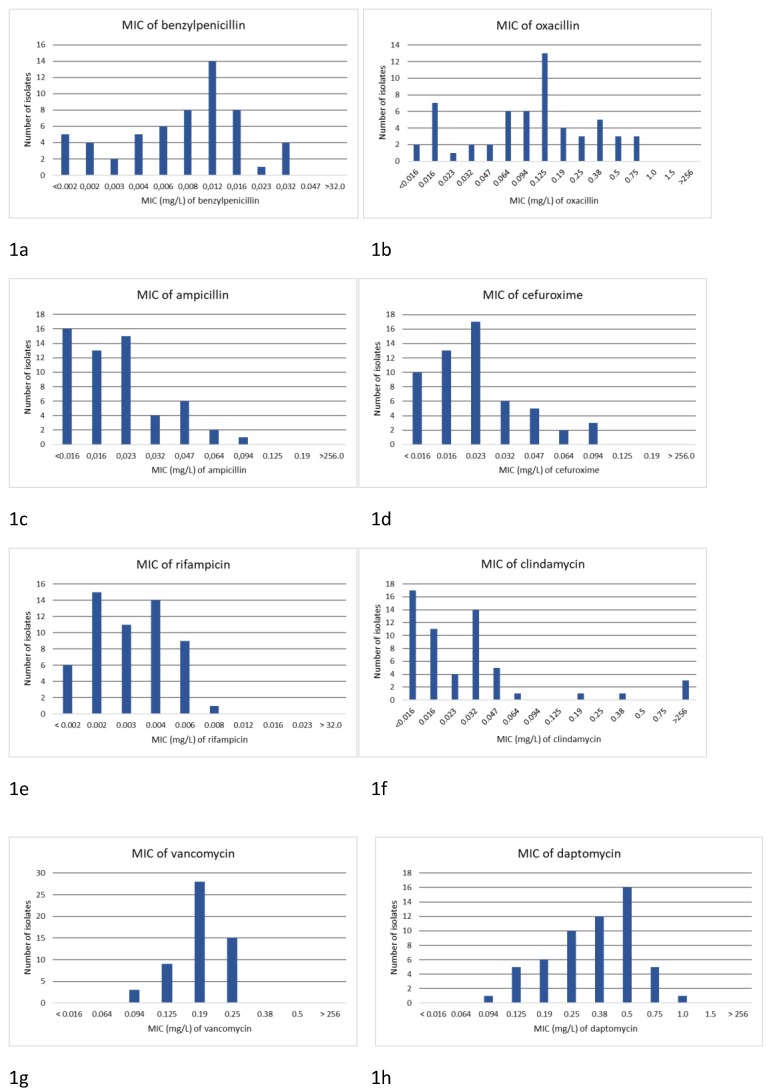
Distribution of MIC values determined by Etest for 57 isolates of *Cutibacterium acnes* obtained from orthopedic implant infections of the knee joint (n=5), hip joint (n=21), and shoulder joint (n=31) for; a) benzylpenicillin, b) oxacillin, c) ampicillin, d) cefuroxime, e) rifampicin, f) clindamycin, g) vancomycin, and h) daptomycin.

The distributions of MIC values for the nine antimicrobial agents are presented in Figures 1a‑i. In general, the MIC values for the beta-lactam antibiotics were low and showed a wild type MIC distribution pattern. The lowest MIC values were noted for benzylpenicillin, with a MIC_50_ of 0.008 mg/L and a MIC_90_ of 0.012 mg/L. The MIC_50_ and MIC_90_ values for cefuroxime were 0.023 mg/L and 0.047 mg/L, respectively.

The MIC values for clindamycin deviated from the wild type group, and 5/57 (8.8%) of the isolates were resistant. An MIC value of >256 mg/L was noted for 3/57 (5.3%) of the isolates, indicating high-level resistance to clindamycin. The MIC values for rifampicin were low, with an MIC_50_ of 0.003 mg/L, an MIC_90_ of 0.006 mg/L, and none of the isolates displayed decreased susceptibility. Daptomycin had an MIC_50_ of 0.38 mg/L and an MIC_90_ of 0.5 mg/L, while vancomycin had an MIC_50_ of 0.19 mg/L and an MIC_90_ of 0.25 mg/L.

**Figure 2:**
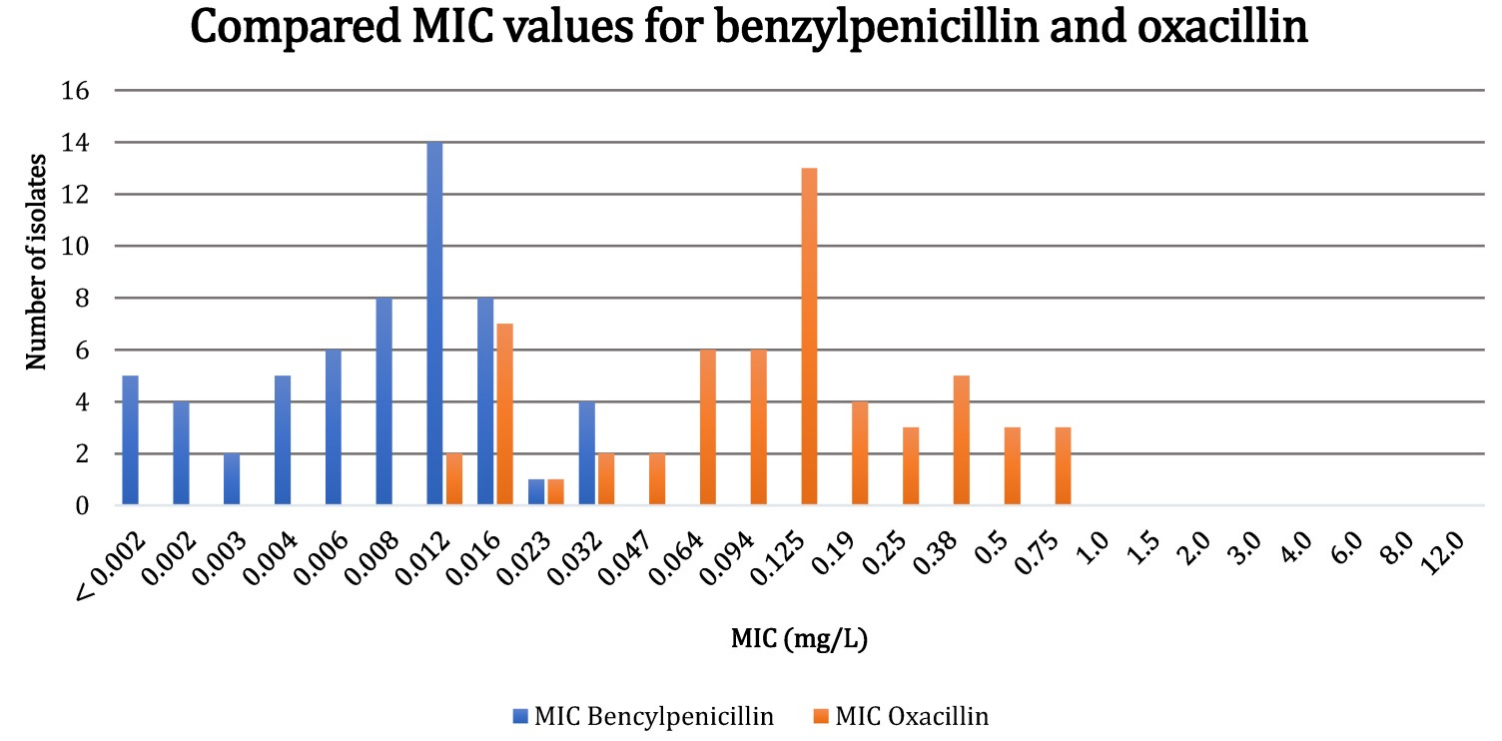
Comparison of MIC values determined by Etest for benzylpenicillin and oxacillin of 57 isolates of *Cutibacterium acnes* obtained from orthopedic implant infections. The lowest MIC values for benzylpenicillin are < 0.002 mg/L and those for oxacillin are < 0.016 mg/l. Due to the different scales on the Etest strips, the lowest value for oxacillin (< 0.016 mg/L) is marked in the figure as 0.012 mg/L. The real MIC values for these strains are assumed to be 0.012 mg/L or lower, but cannot be measured due to the scale on the Etest strips.

Figure 2 provides a comparison of the MIC values for oxacillin and benzylpenicillin. For oxacillin, the MIC_50_ was 0.125 mg/L and the MIC_90_ was 0.19 mg/L. All 57 strains showed high susceptibility to both oxacillin and benzylpenicillin. However, MIC values ≤ 0.016 mg/L for benzylpenicillin and oxacillin were found in 51/57 (89.5%) and 9/57 (15.8%) of the isolates, respectively. The MIC values for benzylpenicillin were 3-4-fold lower when compared to oxacillin.

## Discussion

Since *C. acnes* can be found in the sebaceous glands in human skin 6,7,9,10 and is not eradicated by surface disinfection 11,12,13, optimal prophylactic antibiotic treatment is of great importance during implant surgery. National guidelines from a Swedish national expert group recommend isoxazolyl- penicillin (cloxacillin) as prophylactic antibiotic treatment for joint surgery patients in Sweden (PRISS Expert Group 2; http://lof.se/patientsakerhet/vara- projekt/rekommendationer/). This recommendation is indicated for knee and hip surgery, but hitherto has also been applied to shoulder implant surgery.

Due to the high number of shoulder PJIs caused by *C. acnes*, some departments and orthopedic surgeons have added benzylpenicillin to the isoxazolyl- penicillin prophylactic treatment or replaced isoxazolyl-penicillin with clindamycin. This double prophylactic treatment is based on clinical experience, but no study on this subject has been published so far.

When comparing the MIC values of *C. acnes* obtained from implant-associated infections, we found that benzylpenicillin displayed 3-4-fold lower MIC values than isoxazolyl penicillin. These results indicate that benzylpenicillin should be evaluated as an addition to isoxazolyl-penicillin, if used as anti-staphylococcal prophylactic treatment for shoulder arthroplasty.

A higher dose of beta-lactam antibiotics is associated with an increased risk of adverse events such as gastrointestinal and nephrotoxicity. The risk of these complications should be evaluated and taken into consideration when changing prophylactic regimens.

The clinical outcome of antimicrobial prophylaxis is not solely based only on the MIC value; other factors such as tissue penetration, pharmacokinetics and pharmacodynamics should also be considered 14. Benzylpenicillin and isoxazolyl-penicillin are bactericidal antibiotics, which means that their effect is correlated with the length of time that the antibiotic concentration exceeds the MIC value. The half-life of benzylpenicillin is 30-50 minutes and the serum protein binding is approximately 65%, while the half-life of cloxacillin is 30 minutes and the serum protein binding is 94-98%. Since these antibiotics are bactericidal, the half-life is short, and the serum protein binding is high, the doses administered must be high and given repeatedly during the operating day to achieve an appropriate effect and maintain time above MIC.

All of the 57 *C. acnes* isolates investigated in this study had MIC values for benzylpenicillin of 0.032 mg/L or lower. However, these are *in vitro* data, which is a limitation of the study. To evaluate the clinical effect of prophylactic benzylpenicillin, there is a great need for either register-based and/or controlled randomized clinical studies.

In the present study, MIC values were also determined for other antibiotic agents: ampicillin, cefuroxime, clindamycin, rifampicin, daptomycin, and vancomycin. Clindamycin was the only antibiotic agent with high MIC values for some isolates, with some exhibiting a high level of resistance. Similar results have been shown in previous studies 8,12,15,16,19. This reinforces the fact that *C. acnes* can develop resistance against antibiotic agents. Clindamycin is used as prophylactic treatment for patients with penicillin allergy. Furthermore, selected *C. acnes* isolates can develop resistance to clindamycin 15,16,17,18,19. These factors should be taken into account when selecting clindamycin for surgical prophylaxis.

A limitation of the present study is that cefazolin was not assessed, since it is used as perioperative antimicrobial prophylaxis in many countries 2. However, cefuroxime was assessed and was associated with low MIC_50_ and MIC_90_; the MIC values were 1 - 2 fold higher compared to benzylpenicillin. In addition, cephalosporins have a broader antimicrobial spectrum which may have negative ecological effects. The half-life of cephalosporins is longer when compared to penicillins, which affect the timing and frequency of administration 20.

The isolates investigated in the present study all showed low MIC values for rifampicin (≤0.008 mg/L), and none of the tested isolates were resistant. However, monotherapy with rifampicin usage can select for antibiotic resistance among skin isolates of staphylococci. Since staphylococci are the second most frequent pathogen in shoulder PJIs, rifampicin monotherapy should not be used in a prophylaxis , but rather be reserved as a treatment option for an established PJI. It has also been shown that *C. acnes* has the ability to develop resistance against rifampicin in the same manner as staphylococci; that is, by specific point mutations, which again makes rifampicin monotherapy an inappropriate prophylactic treatment 21.

According to EUCAST (eucast.org), the only specific MIC breakpoints (epidemiological cut-off values) noted for *C. acnes* are 0.125 mg/L for benzylpenicillin and 2.0 mg/L for vancomycin. Daptomycin could also be considered as a prophylactic agent, since it is active against staphylococci including multi-drug resistant *Staphylococcus epidermidis*. However, as of yet there are no reports on the use of daptomycin for prophylaxis in implant surgery.

## Conclusion

The MIC values for benzylpenicillin were 3-4-fold lower when compared to cloxacillin. These *in vitro* results indicate that benzylpenicillin might be a more effective drug to prevent shoulder PJIs caused by* C. acnes*, However, further studies on the subject are needed, and the efficiency and safety of the prophylactic combination therapy should be evaluated using a randomized controlled clinical trial and/or register-based studies.
